# Vegan diet and the importance of nutritional self-concept in the treatment of unipolar depression: a new perspective

**DOI:** 10.1192/j.eurpsy.2025.1354

**Published:** 2025-08-26

**Authors:** C. A. Penkov, K. Friedrich, J. Krieger, M. Ziegenbein

**Affiliations:** 1 Wahrendorff Klinikum, Sehnde, Germany

## Abstract

**Introduction:**

Studies on the psychological impact of a vegan diet and its effect on mental health are still, although the interdisciplinary literature points to significant diet-related cognitions associated with the active choice of a plant-based diet. This study addresses this research gap by framing veganism as an identity-associated aspect of self-concept in young vegans and examines the influence of self-esteem resulting from the vegan diet on symptoms of unipolar depression in a biopsychosocial framework model.

**Objectives:**

veganism as an identity-associated aspect of self-concept

influence of self-esteem resulting from the vegan diet on symptoms of unipolar depression

alternative perspective on the connections between psyche and nutrition

**Methods:**

In a representative sample of n = 659 students from German universities, the absolute and additional influence of diet-related self-esteem on depressive symptoms was investigated using hierarchical regression, taking biopsychosocial covariates into account.

**Results:**

It was found that the self-esteem experience of the test subjects specifically gained from the vegan diet exerts a statistically significant influence on depressive symptoms (B = - 37, SE(B) = 0.02, p <.001) and can also explain a statistically significant additional proportion of the total variance in a biopsychosocial model of depression (ΔR2 = .18, F [1,649] = 272.34, p <.001). Together, the model of eight covariates and nutrition-related self-esteem can explain 57% of depressive symptoms (R2 = .57, F [9,649] = 94.81, p < .001¸ f2 = 0.13). This statistically significant influence of diet-related self-esteem also persists in an exploratory study of different severity levels of depressiogenic distress

**Conclusions:**

The results provide evidence of a psychological impact factor in relation to a vegan diet and identify psychological consequences and thus open up a new research perspective in clinical psychology.

**Disclosure of Interest:**

None Declared

